# Experiences and lessons learned for planning and supply of micronutrient powders interventions

**DOI:** 10.1111/mcn.12494

**Published:** 2017-09-29

**Authors:** Claudia Schauer, Nigel Sunley, Carrie Hubbell Melgarejo, Christina Nyhus Dhillon, Claudia Roca, Gustavo Tapia, Pragya Mathema, Shelley Walton, Ruth Situma, Stanley Zlotkin, Rolf DW Klemm

**Affiliations:** ^1^ Centre for Global Child Health and Home Fortification Technical Advisory Group (HF‐TAG) Toronto Ontario Canada; ^2^ Sunley Consulting Johannesburg South Africa; ^3^ Strengthening Partnerships, Results, and Innovations in Nutrition Globally Arlington Virginia USA; ^4^ The Manoff Group Washington, District of Columbia USA; ^5^ Independent Consultant Geneva Switzerland; ^6^ John Snow, Inc. Guatemala City Guatemala; ^7^ Independent Consultant Cochabamba Bolivia; ^8^ UNICEF Abuja Nigeria; ^9^ Friedman School of Nutrition Science and Policy Tufts University Boston Massachusetts USA; ^10^ UNICEF New York New York USA; ^11^ Center for Global Child Health Hospital for Sick Children Toronto Ontario Canada; ^12^ Departments of Pediatrics & Nutritional Sciences University of Toronto Toronto Ontario Canada; ^13^ Dalla Lana School of Public Health University of Toronto Toronto Ontario Canada; ^14^ Helen Keller International Washington District of Columbia USA

**Keywords:** assessment of nutritional status, infant and child nutrition, iron deficiency anaemia, micronutrients, policy, programme components

## Abstract

Realistic planning for a nutrition intervention is a critical component of implementation, yet effective approaches have been poorly documented. Under the auspices of “The Micronutrient Powders Consultation: Lessons Learned for Operational Guidance,” 3 working groups were formed to summarize experiences and lessons across countries regarding micronutrient powders (MNP) interventions for young children. This paper focuses on programmatic experiences in the planning stages of an MNP intervention, encompassing assessment, enabling environment and adaptation, as well as considerations for supply. Methods included a review of published and grey literature, key informant interviews, and deliberations throughout the consultation process. We found that assessments helped justify adopting an MNP intervention, but these assessments were often limited by their narrow scope and inadequate data. Establishing coordinating bodies and integrating MNP into existing policies and programmes have helped foster an enabling environment and support programme stability. Formative research and pilots have been used to adapt MNP interventions to specific contexts, but they have been insufficient to inform scale‐up. In terms of supply, most countries have opted to procure MNP through international suppliers, but this still requires understanding and navigating the local regulatory environment at the earliest stages of an intervention. Overall, these findings indicate that although some key planning and supply activities are generally undertaken, improvements are needed to plan for effective scale‐up. Much still needs to be learned on MNP planning, and we propose a set of research questions that require further investigation.

## 1 INTRODUCTION

Meeting the nutritional needs of infants and young children in low‐income settings is difficult due to the high costs, low availability, and poor access to nutrient‐rich foods (Dewey, 2013). Since 2011, the World Health Organization (WHO) has recommended micronutrient powders (MNP), a mixture of vitamins and minerals delivered in single‐dose sachets, which are stirred into a child's portion of food immediately before consumption (WHO, 2016). As of 2014, 50 countries were implementing MNP interventions: 9 at the national level and 20 at the subnational level (UNICEF, 2015). Many were also planning to start or expand MNP interventions, with 22 new countries planning to scale up nationally.

Despite the recent expansion of MNP adoption, MNP interventions are not always planned for successfully. According to UNICEF, only one fifth of the 15 million children targeted globally were reached with MNP in 2014 (UNICEF, 2015). Proper planning is important for the successful initiation and scale‐up of nutrition interventions. A well‐designed approach to any programme will diagnose the problem, set goals, select and integrate interventions, and identify resources (Austin & Zeitlin, 1981). The processes required to support these activities, however, often are not transparent, documented, deliberate, or evident in nutrition interventions (Pelletier et al., 2012). New or at‐scale efforts to integrate MNP into ongoing services would benefit from reviewing lessons learned and identifying gaps from country experiences.

This paper is part of a series commissioned by the U.S. Agency for International Development (USAID) through the Strengthening Partnerships, Results, and Innovations in Nutrition Globally (SPRING) project to document experiences in planning, implementing, and monitoring MNP interventions focused on young children and interpret implications for programmes globally. This paper examines MNP planning, with a focus on assessment, enabling environment, and adaptation, as well as supply.

## 2 METHODS

A consultative group consisting of 49 practitioners with knowledge in the implementation of MNP interventions was formed. The process is described in the executive summary of this series (Nyhus Dhillon et al., 2017). Briefly, under the auspices of “The Micronutrient Powders Consultation Lessons Learned for Operational Guidance,” three working groups (WGs) were established: planning and supply (WG1); delivery, social, and behaviour change communication, and training (WG2); and monitoring, process evaluation, and supportive supervision for continual program improvement (WG3). The focus of the consultation was to review interventions that were fairly well established and scaled, targeting children 6–23 months of age. However, as the consultative process unfolded, learnings from pilots and programmes with a wider target age (up to 59 months of age) were included, as well as some relevant lessons from emergency settings.

Each WG was charged with synthesizing available evidence from programmatic settings. The outcomes of this effort are presented in this paper for WG1 and elsewhere in this series for WG2 (Reerink et al., 2017) and WG3 (Vossenaar et al., 2017). WG1 consisted of a chair (RDWK) and 14 participants working for governmental institutions, multilateral and international organizations, universities, and independent consultants. WG members were based in Bolivia, Canada, Guatemala, Nigeria, South Africa, and the United States. WG1 participated in a yearlong (July 2015–July 2016) consultative process. It held four teleconferences to define the scope of the WG topic; participated in a meeting on October 19 and 20, 2015, in Washington, DC, United States; exchanged emails; conducted key informant interviews; and reviewed literature.

The WG obtained primary data from key informants identified using purposive and snowball sampling (Table 1). Key informants either completed a questionnaire or were interviewed using the same structured questionnaire (Supporting Information S1). Follow‐up with key informants to confirm data and seek additional information was performed as necessary. WG members involved in implementation also completed questionnaires or were interviewed. Data were analysed by collating the information into a spreadsheet and identifying relevant information. We also identified key informants to provide information for case studies to take a more in‐depth look at context‐specific learning. Key informants provided expert opinion as part of their professional capacity and regular public health practice. Thus, the activities involved in the consultation did not meet the human subjects' research definition and were considered exempt by the John Snow, Inc. Institutional Review Board. Interview participants were told their names would be confidential in all reports and manuscripts and that the information from this consultative process would be summarized in manuscripts submitted for peer review publication.

**Table 1:** Characteristics of key informants^a^

Key informant number	Country(ies) of work^b^	Role of informant	Scale of programme(s)^c^	Data collection method	Interview date
1	Kyrgyzstan, Mozambique, Nepal, Niger, Uganda	TA provider	National, subnational, pilot	Interview	September 15, 2015
2	Madagascar	Implementer	Pilot	Interview	September 24, 2015, September 30, 2015
3	Kyrgyzstan	TA provider	National	Interview Questionnaire	September 24, 2015, and October 10, 2015
4	Multiple	TA provider	N/A	Interview	October 5, 2015
5	Multiple	Supplier	National, subnational, pilot	Interview	October 7, 2015
6	Ethiopia, Kyrgyzstan, Madagascar	TA provider	Pilot, national	Interview	October 8, 2015
7	Bolivia	Policymaker	National	Questionnaire	October 9, 2015
8	Afghanistan, Bangladesh, El Salvador, Kenya, Nigeria	TA provider	National, subnational, pilot	Questionnaire	October 11, 2015
9	Tanzania	Implementer	Pilot	Questionnaire	October 12, 2015
10	South Sudan	Implementer	Pilot	Questionnaire	October 13, 2015
11	Multiple	TA provider	National, subnational, pilot	Questionnaire	October 13, 2015
12	Bangladesh Mozambique, Pakistan	TA provider	Pilot	Interview	October 13, 2015
13	Multiple	Supplier	N/A	Interview	October 13, 2015
14	Cambodia	Implementer	Pilot	Questionnaire	October 14, 2015
15	Liberia, Nepal, Nigeria	TA provider	Subnational, pilot	Interview Case Study	October 16, 2015, and July 11, 2016
16	Mozambique	Policymaker	Pilot	Questionnaire	October 23, 2015
17	Colombia	Supplier	N/A	Interview	November 2, 2015
18	Guatemala	Supplier	National	Interview	November 2, 2015
19	Bangladesh	Implementer	National	Questionnaire	March 22, 2016
20	Bolivia	TA provider	National	Case Study	June 21, 2016
21	Uganda	Implementer	Pilot	Case Study	July 11, 2016

a TA, technical assistance.

b Defined on the basis of the primary countries for which key informant provided experiences and learning, unless a key informant was a supplier or worked extensively in more than five countries.

c Defined by the stage of the intervention key informant provided experiences and learnings.

The WG obtained secondary data from a systematic search of peer‐reviewed and grey literature. The search inclusion criteria was implementation learning on MNP from database inception through December 2015 and included a screening of abstracts, along with full texts when required, as described in more detail in the executive summary of this series (Nyhus Dhillon et al., 2017). A broad interpretation of relevance was applied when selecting literature to maximize the potential secondary data.

This paper is divided into two sections: planning and supply. We borrow the framework proposed by Meyers, Durlak, and Wandersman (2012) on quality implementation to structure the planning section into three subthemes: assessment, enabling environment, and adaptation. According to this framework, the initial considerations for a new intervention comprise assessment, capacity‐building strategies, and decisions about adaptation (Meyers et al., 2012). Assessment was defined as a means to determine the degree of fit between the new intervention and the setting. The upstream capacity‐building strategy was defined as “obtaining explicit buy‐in from critical stakeholders and fostering a supportive community/organizational climate,” which includes engaging opinion leaders, aligning to policies, removing barriers to use, and identifying advocates for the proposed intervention. We broadly consider this the “enabling environment” and do not consider in this paper the more downstream implementation‐level capacity (organizational and staff capacity, etc.) identified in the Meyers framework. Adaptation was defined as identifying elements that need to be retained and those that can be modified to the context. The framework was not explicit with regard to financing, so we placed this after the adaptation section.

Although some planning considerations are relevant only at start‐up, many must be reconsidered for potential scale‐up as part of regular ongoing decisions about programme adaptation. We used the paper by Gillespie, Menon, and Kennedy (2015) to guide our thinking on scale‐up. They identify nine elements that are useful for scaling up nutrition interventions, and our paper touches on most of them: (a) having a clear vision; (b) intervention characteristics, that is, what is being scaled; (c) context for scaling up; (d) drivers and barriers; (e) contextually relevant strategies and pathways for scaling up; (f) capacity to scale up; (g) ensuring adequate governance structures and systems; and (h) ensuring adequacy, stability, and flexibility of financing. The ninth element—monitoring, evaluation, learning, and accountability—is discussed in the WG3 paper (Vossenaar et al., 2017).

The findings from this review are presented as a series of statements that relate to current practice, followed by details of the findings from countries on which these statements are founded. This analysis is not designed to provide results from any individual country. Terms and working definitions for the content of this paper, defined on the basis of literature and key informants, are presented in Box 1. The authors acknowledge that other definitions may apply outside the context of this paper.

**Box 1: Definitions of terms used in programmatic research by working group 1 (WG1): Planning and supply^a^**

**Acceptability**: An approach or product being well‐received culturally, socially, and per organoleptic and similar subjective factors
**Advocacy**: Presenting stakeholders with evidence‐based information to help them decide in favour of the intervention
**The Code of Marketing of Breastmilk Substitutes (“the Code”)**: A WHO‐endorsed international policy framework to protect breastfeeding from the inappropriate promotion of commercial products
**Cost sustainability**: Pricing and delivery approach (public vs. private and who pays) that allows costs to be sustainably covered
**Feasibility**: Evidence that an approach will function properly in terms of logistics, climate, timing, economics, and similar factors
**Formative research**: A process that occurs before an intervention to “obtain detailed information about the people for whom, and the context in which, interventions will be designed” (Gittelsohn et al., 2006)
**Integration**: Including an MNP intervention in existing policies or programmes so that MNP may benefit from existing infrastructure
**Intervention**: An activity or product within a programme, such as MNP within a larger effort on IYCF
**Assessment**: A review of relevant data, including micronutrient status, dietary gaps, and existing policies and interventions, used to assess appropriateness of MNP and countries' readiness and opportunities
**Regulatory environment**: Laws and guidelines governing how pharmaceutical and food products can be manufactured, imported, or distributed
**Reliable supply**: Uninterrupted, continuous, quality MNP supplies delivered to the right person, at the right time, in the right way
**Scale‐up**: Expand efficacious interventions to more people over a wider area, maintaining the same quality, equity, and sustainability
**Supply management**: Procuring and handling (tracking, transportation, storage, and integrating with existing supply chains) for constant availability of sufficient high‐quality, safe, and consumable MNP (in the proper delivery channels and locations)

^a^IYCF, infant and young child feeding; MNP, micronutrient powder; WHO, World Health Organization.

## 3 RESULTS

Sixty‐six peer‐reviewed articles, 16 guidance documents, and 45 programme reports or conference presentations with information on implementation experiences were identified and reviewed (Nyhus Dhillon et al., 2017). Fifty‐one documents were identified as relevant for planning and supply. Twenty‐one key informants were interviewed, completed a questionnaire, or participated in the development of case studies (Table 1). Lessons from 19 countries in all six WHO geographic regions were considered, some with multiple experiences with MNP pilots and programmes.

Case studies from Bolivia (Box 2; KI 20), Nepal (Box 3; KI 15), and Uganda (Box 4; KI 21) illustrate key experiences. Those three experiences highlight lessons learned at different stages of planning. Bolivia was a national government effort from the beginning, with MNP replacing another commodity already in use (KI 20), whereas Nepal started with a limited pilot that was used to plan and scale up (KI 15). Uganda was in the early planning and piloting phase (KI 21).

**Box 2: Bolivia case study^a, b^**

**Table:** 

Where	National level
When	Intervention started on a national scale in 2006 and is ongoing
Partners	The Bolivian MSD leveraged an existing public health care system already distributing iron syrup, to transition to MNP in 2006.
Objectives	To integrate MNP (branded as *Chispitas*) into the country's Zero Malnutrition Program, a multisectoral programme implemented by the Government of Bolivia and its partners to eradicate malnutrition in children under 2 and to decrease malnutrition in children under 5.
Target population	The MNP intervention initially targeted all children 6–23 months. In 2013, the intervention was expanded to all children under 5.
Coordination	Multiple ministries, including the MSD, formed the National Committee on Food and Nutrition in support of implementing Zero Malnutrition Program. Their mandate was to plan and implement multisectoral strategies and mobilize funding and technical assistance from national and international stakeholders.
Enabling environment	In 2003, the Bolivian national demographic and health survey showed that 60% of children under 5 and 72% of children under 2 were anaemic. In 2005, a collaborative group (e.g., MI, PAHO, UNICEF, and WFP, working with MSD) reviewed possible options for anaemia control. With their findings, the MSD decided to replace iron syrup with MNP for all children 6–23 months. MNP was included as a benefit within Bolivia's social protection package, the universal maternal‐child insurance fund (SUMI). This ensured that procurement and basic distribution and delivery costs were absorbed and embedded within SUMI.
Evidence generated	A randomized controlled trial was conducted by Bolivian researchers under similar circumstances to those of the former intervention for the prevention and control of anaemia in children 6–23 months. From this study, the researchers concluded that the use of MNP increased adherence to treatment and significantly reduced rates of anaemia compared to ferrous sulfate syrup (Urquidi, Mejía, & Vera, 2009).
Supply issues	MNP was registered with the MSD as a supplementary food. The MI donated the initial 6 million sachets (for 100,000 children).
	In 2008, MSD issued a request for tender to Bolivian pharmaceutical manufacturers to provide a national supply of *Chispitas*. Stock‐outs were frequently experienced and due primarily to factors including inaccurate forecasting, order delays due to limited supplier capacity, and delays receiving SUMI funds for MNP procurement.
Outcomes	By 2013, MNP coverage reached 72% of approximately 536,000 children 6–23 months of age. A nationally representative survey found 74% of urban caregivers and 82% of rural caregivers demonstrated adequate preparation of MNP. As a measure of adherence, 45% of urban and 52% of rural caregivers reported that children consumed all 60 sachets.
Lessons learned	The integration of MNP into existing public health and nutrition programmes is a feasible approach to enable large‐scale distribution. Support for the scale‐up process can be enhanced by government agencies and policymakers who include MNP within national development plans and give prioritization to multisectoral coordination, engagement of the private sector, and resource mobilization.
	Once a programme can generate sufficient orders, and demand for the product is steady, local manufacturing can be a reliable and cost‐efficient approach to maintaining a quality supply.

MNP, micronutrient powders; MI, Micronutrient Initiative; MSD, Bolivian Ministry of Health and Sports; PAHO, Pan‐American Health Organization; SUMI, Universal Maternal‐Child Insurance Fund; UNICEF, United Nations Children's Fund; WFP World Food Programme.

Based on information from key informant 20

**Box 3: Nepal case study^a, b^**

**Table:** 

Where	Currently rolled out to 20 districts covering one third of the country.
When	The intervention started with a small‐scale feasibility study on MNP distribution linked with IYCF in 2009, which led to an implementation of large‐scale pilot in six districts to design an approach to scale up the intervention nationally.
Partners	The initiative has been led by the MoH with lead support from UNICEF‐Nepal. The program design and implementation have also been supported by the CDC, the National Planning Commission, the Institute of Medicine, WFP, and Micronutrient Initiative. Funding was provided by the European Union, Australian Aid, Work Bank, International Zinc Association, UNICEF, and the Government of Nepal.
Objectives	The main goal of the IYCF‐MNP (“Baal Vita”—Vitamins for Children in Nepali) program is to reduce anaemia in young children by ensuring more than 80% of 1.5 million children 6–23 months of age consume a course of MNP twice a year by progressively scaling up the intervention nationally by 2017. The program also aims to promote optimum feeding practices to improve growth of 3.7 million children under 5.
Target population	The primary target for the national program is children 6–23 months of age. In emergency settings, such as general food ration distribution in food‐insecure areas by WFP and in a UNHCR‐supported Bhutanese refugee camp, MNP distribution has covered children under 5.
Coordination	A multistakeholder committee chaired by the National Planning Commission was formed to design the program. MoH coordinated rollout with strong engagement with various stakeholders.
Enabling environment	A national situational anaemia analysis in 2003 underscored the urgent need to address anaemia in children. Almost half of children under 5 and around 75% of children under 2 were suffering from anaemia. In 2005, the National Nutrition Strategy and Anemia Plan of Action endorsed MNP as a key intervention. In 2007, the Joint Stakeholder National Nutrition Priority Workshop approved MNP piloting, with strong emphasis on IYCF promotion.
	With the feasibility study and pilot, the “strategic plan for initiating and scaling up IYCF community promotion linked with MNP” was formulated to serve as the road map. Furthermore, it has also been highlighted as a key intervention in the Multi‐Sector Nutrition Plan for national scale‐up.
Evidence generated	The pilot phase demonstrated that for both health facility and community‐based distribution, female community health volunteers can help achieve strong and equitable coverage and compliance. Regular social mobilization through community‐based organizations is also important for maintaining good performance.
Supply issues	In Nepal, MNP is considered a food supplement. Initially, the product was procured by UNICEF, but over time, the government has started procuring it with its own resources through a Health SWAp pooled funding mechanism. The distribution of MNP and communication materials has been integrated into the government's logistics management system and the reporting of supply status has been institutionalized.
Outcomes	The coverage of MNP distribution has consistently reached over 60% and as high as 83% in the past 5 years of implementation. An evaluation is ongoing to document the impact of the intervention, including its contribution in reducing anaemia and providing guidance to enhance efficiency and effectiveness of the future programming.
Lessons learned	Nepal has adopted a phased approach to initiate and scale up MNP, starting with generating strong acceptance for the intervention through extensive advocacy and stakeholder engagement, and then designing a national approach based on a large‐scale pilot. One of the key factors for success is integrating MNP with IYCF promotion.

CDC, Centers for Disease Control and Prevention; IYCF, infant and young child feeding; MNP, micronutrient powders; MoH, Ministry of Health; SWAp, Sector‐Wide Approach; WFP, World Food Programme; UNHCR, United Nations High Commissioner for Refugees; UNICEF, United Nations Children's Fund.

Based on information from key informant 15

**Box 4: Uganda case study^a, b^**

**Table:** 

Where	Pilot project in different parts of the country
When	Coordination through the newly formed MN‐TWG started in 2012. Formative research began in 2014, and SPRING's distribution began in 2016.
Partners	Private and public sector partners including UNICEF, WFP, the MoH, SPRING, and research partners.
Objectives	To develop harmonized tools and to coordinate pilots in different parts of the country to explore the potential for rollout of MNP in Uganda.
Target population	Children 6–23 months of age
Coordination	In 2012, following a regional UNICEF/CDC MNP workshop, the Uganda MoH initiated the introduction of MNP by establishing the MN‐TWG. This group is composed of representatives from UN bodies (REACH, UNICEF, WFP, and WHO), USAID‐funded projects (Community Connector, SPRING, and Harvest Plus), Uganda Health Marketing Group, Makerere University, and other development partners.
Enabling environment	The MN‐TWG collaborated with many groups within and outside of the MoH. The group participated in the development of national micronutrient guidelines (to ensure there was a policy framework for MNP distribution), formation of a draft implementation framework, and develop a harmonized social behavioral change communication plan and monitoring tools.
Evidence generated	Implementing partners conducted formative research prior to the start of implementation. These findings suggested that although MNP was acceptable in Ugandan communities, sponsorship by local officials and the MOH would be important for continued acceptance. Findings from the pilot are not yet available but will be used to inform national programming and further use of MNP in the country.
Supply issues	In SPRING's experience with MNP procurement, 6 months was required to prepare an appropriate request for proposals for MNP. The request for proposals included references, financial statements, information on formulation, data on overages (micronutrient levels beyond the WHO recommendations), product shelf life, terms of delivery, and payment. After the request for proposals, in Uganda, 14 months was required before the MNP was delivered.
	The U.S. Government considers MNP a pharmaceutical product, which, per ADS 312, is subject to U.S. Government approval and regulations regarding quality and sources (i.e., not local) of the product.
Outcomes	SPRING is conducting surveys to estimate reach/coverage, adherence/use, and cost‐effectiveness. SPRING is also carrying out qualitative work to understand the issues related to the use or nonuse of the product after 2 months. WFP and CDC conducted baseline assessments and will conduct follow‐up work to look at MNP use and anaemia prevalence to understand the effectiveness of MNP in Uganda.
Lessons learned	Procurement processes can be complex, as each implementing partner's procurement processes, in addition to the donor's processes, needs to be followed. Furthermore, there are additional processes that apply to MNP. In Uganda, SPRING learned that developing local packaging for MNP takes a long time, as several stakeholders' inputs need to be resolved (e.g., the International Baby Food Action Network wanted to ensure the product would not be misunderstood as a breast milk substitute).
	Product registration is a time‐intensive process that requires significant support and coordination from the MoH. It was also a lengthy process to develop a policy framework to allow MNP programming. This process required buy‐in from many outside of the MN‐TWG. That buy‐in ultimately helped facilitate a range of follow‐on tasks, including product registration and institutional review board approval for studies.
	Scale‐up was built into the planning from the start. The scale‐up is organized in two stages. In the first stage, MN‐TWG partners undertake implementation research studies on MNP distribution in identified districts to gauge acceptability of MNP and document distribution options in pilot districts. On the basis of lessons from this pilot process, the second step will consist of the national introduction of MNP, led by the MoH in collaboration with other stakeholders.

CDC, Centers for Disease Control and Prevention; MNP, micronutrient powders; MN‐TWG, Micronutrient Technical Working Group; MoH, Ministry of Health; REACH, Renewed Efforts Against Child Hunger; SPRING, Strengthening Partnerships, Results, and Innovations in Nutrition Globally; UNICEF, United Nations Children's Fund; USAID, United States Agency for International Development; WFP, World Food Programme; WHO, World Health Organization.

Based on information from key informant 21

### 3.1. Planning

#### 3.1.1. Assessment

##### 3.1.1..1 Assessments have often been limited in scope

The decision to implement an MNP intervention should be based on an assessment of nutrient gaps, existing policies and programmes to meet these gaps, and readiness to implement a new intervention (Neufeld & Cameron, 2012; WHO, 2016). Such assessments have the potential to determine whether an MNP intervention should be initiated, substantiate an MNP formulation (3, 5, 15, or 22 micronutrients are standard), determine target populations or subregions of the country on which to focus efforts, identify existing related interventions, and consider potential delivery strategies (Neufeld & Ramakrishnan, 2011; KI 4).

Assessments have largely focused on the prevalence of anaemia, and in a few instances micronutrient status and/or adequacy of nutrient intake, to justify an MNP intervention (KIs 1, 2, 3, 4, 6, 7, 9, 10, 11, 12, 14, 15, and 20). In Mongolia, stakeholders decided to introduce MNP based on an assessment that reported a 33% prevalence of vitamin D deficiency, a 32% prevalence of iron deficiency anaemia, and low intake of micronutrient‐rich foods and iron supplements among children (World Vision Mongolia, 2005). Likewise, in Kyrgyzstan, an MNP intervention was initiated on the basis of data showing high anaemia rates, low rates of infections, and poor diet quality (Lundeen et al., 2010). In Bangladesh, micronutrient gaps, and the extent to which food‐based approaches and/or MNP could meet these gaps, were assessed; it was concluded that both intervention strategies should be implemented (Karim et al., 2005). Data on micronutrient deficiencies and MNP efficacy were important for advocacy during both start‐up and scale‐up (Lundeen, Imanalieva, Mamyrbaeva, & Timmer, 2013).

An assessment of existing nutrition activities at the national and local levels benefits from a review of protocols, coverage and demand for existing services, and the quality of those services. This type of information has been useful for identifying entry points and ways to integrate MNP into existing systems (KI 6). For example, in Mexico, MNP replaced a fortified complementary food provided to children 6–23 months of age as part of a larger social protection programme. This decision was based on a series of studies indicating that anaemia might be reduced at a lower cost and with lower risk of chronic illness (given Mexico's nutrition transition, where low levels of stunting, wasting, and underweight were coupled with increasing risk of overweight and obesity; Neufeld et al., 2011).

Key informants underscored the importance of assessing a country's capacity to take on an MNP intervention (e.g., supply chain, staff training, and community mobilization) but noted that MNP assessments tended to neglect this consideration (KIs 2, 9, and 16). Rather, capacity issues were generally not discussed by stakeholders until after the pilot phase (KI 9). In situations where weak capacity was acknowledged, it did not appear to influence the decision to proceed with the intervention (KIs 2 and 16). Further, technical partners tended to play a limited role during the implementation phase, and it was suggested that developing plans for continued learning with input from technical partners would be beneficial (KI 16).

Key informants highlighted the importance of involving a diverse group of stakeholders in the assessment process, including government officials, multilaterals, nongovernment organizations, donors, the private sector, and research institutions (KIs 1, 3, 7, 9, 14, 16, and 20). Involving stakeholders at this early stage has played a particularly important role in promoting buy‐in once the intervention was warranted, as in the case of Uganda (SPRING, 2017).

#### 3.1.2. Enabling environment

##### 3.1.2..1 Generating buy‐in for MNP started at the global level and has been adopted by countries ready to try a new approach

Globally, MNP interventions have gained support in the recent years and were included as part of a package of 13 efficacious and highly cost‐effective interventions to improve the nutrition of young children in a World Bank report (Horton, Shekar, McDonald, Mahal, & Brooks, 2010). This package became the basis for the nutrition interventions within the Scaling Up Nutrition movement, which has helped to strengthen commitment and support country‐led nutrition interventions (Scaling Up Nutrition, 2011). The Home Fortification Technical Advisory Group (HF‐TAG; http://www.hftag.org), a global network of stakeholders who provides technical guidance and best practices on MNP interventions, has played an important role in generating buy‐in. These global efforts are likely the explanation for the recent scale‐up of MNP noted earlier. Participation by country‐level decision makers in global or regional meetings has catalysed countries to introduce an MNP intervention (KIs 15 and 16).

Successful consensus generation for MNP in countries has often occurred because the political environment was ripe for a new intervention. Stakeholders were interested in pursuing approaches that could be effective, as they faced challenges with iron syrup interventions (in Kyrgyzstan and Bolivia) and/or experienced success of large‐scale food fortification (in Kyrgyzstan and Tanzania; KIs 7 and 20). In Nigeria, MNP was introduced when the government and partners in child malnutrition were seeking a shift from treatment to preventive approaches (KI 15).

A common concern about introducing MNP has been the safety of iron interventions, particularly in the context of malaria (Neuberger, Okebe, Yahav, & Paul, 2016). The WHO guidance on implementing MNP concurrently with malaria control measures was useful in addressing this concern (WHO, 2011). In Tanzania, after a review of the WHO policy and research, maintaining a 10‐mg iron dose was agreed upon (KI 9). Similarly, in Nigeria, consensus needed to be reached by health authorities in all 37 states on whether to include MNP in the national micronutrient guideline. After zonal meetings in areas with a high burden of malaria, it was agreed that the benefit outweighed the risk, and the guideline in Nigeria was revised to incorporate a chapter on MNP (KI 15).

Table 2 summarizes messages that have been effective to reach key stakeholders, as identified by key informants, and generate buy‐in.

**Table 2:** Key advocacy messages communicated to stakeholders at initiation and/or scale‐up as described by key informants and literature^a^

Stakeholder audience	Areas of concern	Country‐level experience	Summary of key advocacy messages cited by key informants and given to stakeholders
Health and nutrition experts, including Ministry of Health staff, paediatricians, and pharmacists	Multiple interventions are already available to children, e.g., iron syrup, vitamin A supplementation, and fortified foods on the market. Potential risks of providing iron supplementation in malaria‐endemic settings	Bolivia, Cambodia, Kyrgyzstan, Madagascar, Nigeria, Pakistan, Tanzania	Iron syrup compliance may be low, due to taste and side effects (Urquidi et al., 2009). High‐dose vitamin A supplements to children 6–59 months can exist in combination with MNP where risk of vitamin A deficiency is high.
			Fortified foods have been successful in many contexts and offer an important solution to the general population but might not reach or be adequate for young children.^b^
			Iron‐containing MNP can be given in malaria‐endemic areas, as long as effective malaria prevention and management services are also in place (WHO, 2016).
Agriculture and food security specialists	Potential for MNP to replace efforts to promote local foods	Bangladesh, Cambodia, Tanzania	MNP does not replace local foods and has been used to promote local diets and good IYCF practices.
			MNP can increase the consumption of local staple foods.
Policymakers, including Ministry of Finance and Ministry of Health staff who allocate funds	Cost‐effectiveness of MNP over other interventions addressing anaemia. Cost of adding another public health intervention and ability to afford new intervention long term	Cambodia, Kyrgyzstan, Nigeria, Pakistan	Micronutrient interventions such as MNP are cost‐effective, e.g., $37 in earnings from $1 invested (“Copenhagen Consensus: Expert Panel Findings,” 2012; Horton et al., 2010; Sharieff et al., 2006). Tanzania used local cost‐effectiveness data showing an estimated 3% loss of GDP due to micronutrient deficiencies (Verster, 2010).
			MNP can be distributed through existing channels for cost savings.
			Iron supports cognitive and physical development, ultimately contributing to a country's GDP.
Donors	Overburdened public health systems and challenges in providing access to free health interventions, especially among hard‐to‐reach populations. Capacity to establish complementary public and private distribution channels Sustainability and self‐financing by the country after the initial donor investments end Use in humanitarian and emergency contexts	Global	Supporting delivery mechanisms that are feasible and scalable and have the potential to become self‐sustaining.
			Health system distribution has been complemented by market‐based approaches.
			In humanitarian response, consider supporting the supply short‐term as part of the emergency package with the long‐term vision of integrating into development plans.
Government regulators, advocates of the International Code of Marketing of Breastmilk Substitutes	Ambiguity around registering as a food product or a pharmaceutical Inappropriate promotion of foods for infants and young children	India, Madagascar, Tanzania	MNP can be registered as either a pharmaceutical or food product, based on a country's regulatory framework.
			MNP can also be considered a food fortificant where standards apply for nutrient levels, safety, and quality, and where messaging does not promote use of the product for infants under 6 months.
**Documents indicated as useful for advocacy**:			
DHS, MICS, and similar high‐quality surveys			
Cochrane Review and other systematic reviews (De‐Regil et al., 2013; Dewey, Yang, & Boy, 2009)			
Joint statement from WHO, WFP, and UNICEF regarding micronutrient deficiencies in emergencies (WHO/WFP/UNICEF, 2007); Guideline on the Use of Multiple Micronutrient Powders for Home Fortification of Foods (WHO, 2016)			
Programmatic Guidance Brief on Use of Micronutrient Powders for Home Fortification (World Food Programme et al., 2011)			
Studies showing that alternatives (e.g., iron syrup) have adherence issues and are likely ineffective (MacLean et al., 2013; Urquidi et al., 2009)			
Studies of efficacy (under ideal conditions) and effectiveness (real‐world conditions), as well as results from own or other countries (Giovannini et al., 2006; Jack et al., 2012)			
Copenhagen Consensus, 2012			
MNP Toolkit to Support Countries in Implementing MNP programmes (UNICEF, et al., 2016)			

a CDC, Centers for Disease Control and Prevention; DHS, Demographic & Health Survey; GDP, gross domestic product; HF‐TAG, Home Fortification Technical Advisory Group; IYCF, infant and young child nutrition; MICS, Multiple Indicator Cluster Survey; MNP, micronutrient powders; UNICEF, United Nations Children's Fund; WFP, World Food Programme; WHO, World Health Organization.

b As the interviews were held and this statement was made, the 2016 WHO MNP guidelines state (p. 6): “If sugar is fortified with vitamin A, vitamin A should be excluded from the multiple micronutrient powders. If other staple foods regularly consumed by children (e.g. oil) are fortified with vitamin A, the risk of inadequate and high intakes of vitamin A should be assessed and the decision to include or exclude vitamin A from the multiple micronutrient powders should be based on that assessment prior to programme implementation, with regular review to permit adjustment of vitamin A as needed.” (WHO, 2016)

##### 3.1.2..2 Country‐level leaders and committees have led coordination and advocacy efforts

Often, existing nutrition technical committees or advisory groups, usually government‐led, have subsumed MNP into their mandate or developed MNP‐specific subgroups. Madagascar's National Task Force on IYCF, Cambodia's Nutrition Working Group, and Kyrgyzstan's Technical Working Group all took leadership on MNP. Tanzania's National Food Fortification Alliance created an MNP subgroup (KIs 2, 3, 9, and 14). In some cases, a new group has been formed to support MNP; for example, Mozambique formed an MNP Working Group and Uganda formed a Micronutrient Technical Working Group (KIs 9 and 16). Regardless of the membership or structure, it has been important to establish lines of responsibility and coordination on oversight, progress review, and course correction (Kodish, Rah, Kraemer, de Pee, & Gittelsohn, 2011).

Although these coordinating bodies have largely been successful in building credibility, facilitating advocacy, and providing MNP planning and oversight, their efforts have not always been sufficient. Coordination bodies should also ideally mobilize resources, maintain accountability, strengthen capacity, pursue sound technical design, and assess the ongoing interventions (Pelletier & Pelto, 2013; UNICEF, CDC, & HF‐TAG, 2016). To complement the predominantly technical focus that most country committees have adopted, constituting or engaging a second body focused on political commitment has been useful if there is good communication fed up from the technical arm (KI 6). For instance, in Kyrgyzstan, it was found that functional decision makers, such as parliamentarians, played an important role in the allocation of resources, separate from their technical counterparts (KI 6). Countries have also increasingly recognized the importance of local‐level coordination and input, such as engaging municipal councils (in Bolivia; KI 7) or holding regular meetings with health centres and volunteer health workers (in Cambodia; KI 14).

##### 3.1.2..3 Aligning and integrating MNP into existing policies and programmes has been useful for scale up

Effective planning for eventual scale‐up has often included integrating MNP into existing policies, and this has been relatively successful (KIs 2, 3, 4, 6, 7, 14, 16, 20, and 21). As of 2014, 44 countries had identified MNP interventions in their national nutrition strategies, and 16 had included the product in their national list of essential commodities (UNICEF, 2015). Policy development has generally been an ongoing process that occurs at varying time points. For instance, in Cambodia, policy development ran parallel to pilots or studies being implemented (KI 14), whereas in Kyrgyzstan, policy was put into place after major implementation began (KI 3), and in Nepal, MNP policy was established prior to implementation (KI 15). Without policies in place, some MNP interventions have experienced reduced access to delivery platforms and financial support, limiting scale‐up and sustainability (MacDonald & Altengeral, 2011).

Integration of an MNP intervention into existing programmes has been more of a challenge, although all key informants felt it was crucial if not essential. Integration within the reproductive, maternal, newborn, and child health package of interventions along the continuum of care model conceptually fits with services to promote IYCF (KIs 1, 4, 6, 14, 15, and 16). However, many IYCF programmes still require capacity‐building themselves (KIs 1 and 6). This can impede programmatic quality and coverage (Neufeld & Cameron, 2012), as discussed further in the delivery paper of this series (Reerink et al., 2017). Although only 22% of MNP interventions were classified as “standalone” according to the United Nations Children's Fund (UNICEF) NutriDash (2015), interventions need to be better integrated into existing systems (supply, monitoring, supervision, etc.) to avoid becoming a series of pilots that are not scaled up nor sustained. The ongoing integration challenge seems to lie in the transition from pilots, which can be only integrated to a limited degree (provisionally by geographical area), to scale up, where integration needs to be built into policies and systems at the national or subnational level.

#### 3.1.3. Adaptation

##### 3.1.3..1 The MNP interventions have often focused on children 6–23 months of age, including targeting the most vulnerable children in small geographic areas

The MNP interventions reviewed generally focused on “blanket” distribution to children 6–23 months of age. In some cases, there have been plans to expand to 24–59 months, including potentially selling MNP for the older age group. In Cambodia, where government‐funded blanket distribution of MNP for children 6–23 months of age was seen as an unrealistic (and perhaps unnecessary) goal, Helen Keller International, UNICEF, and other partners focused on securing financial and institutional commitments from government to purchase a regular supply of MNP to support targeted distribution to children from the most vulnerable communities. This would include covering the related travel costs of community workers distributing MNP in hard to reach areas (KI 14). Similarly, in Tanzania, target areas were based on the highest rates of stunting, but the population targeted in this subsidized model has been all children 6–59 months (KI 9).

##### 3.1.3..2 Formative research findings provide information on local context, behaviours, and perceptions

Typically, formative research for MNP has centred around product packaging, messaging, acceptability, and feasibility. Methodologies have generally included ethnographic studies (Pelto, Armar‐Klemesu, Siekmann, & Schofield, 2013), acceptability trials (Young, Blanco, Hernandez‐Cordero, Pelto, & Neufeld, 2010; Osei et al., 2014; M. E. Jefferds et al., 2010), and feasibility studies (Loechl et al., 2009). The studies have been useful in determining willingness of mothers to add the product to a child's food, household behaviours, acceptability of packet instructions and packaging formats, costs of products, and willingness to pay (de Pee et al., 2007; Kodish et al., 2011).

Formative research has allowed for early learning to identify and resolve potential obstacles and prevent costly mistakes later in the life of the intervention (KIs 1 and 14). In Bolivia, inappropriate use and doubts about the product, which were later difficult to correct, were attributed to the lack of formative research conducted (MacLean, Jalal, Loayza, & Neufeld, 2013). In Nigeria, a formative research phase of 9 months was needed to develop a culturally appropriate behaviour change communication strategy to promote MNP acceptability and utilization among its diverse population (Kodish et al., 2015). Formative research in Kenya alerted programmers not to include anaemia messages because the condition was perceived as a rare and serious illness (Jefferds et al., 2009).

##### 3.1.3..3 Pilots have been important to test programme design and potential for scale

Key informants commented that further efficacy studies are not needed; if MNP are produced and used as directed, they have the effect of reducing anaemia related to iron deficiency (De‐Regil, Suchdev, Vist, Walleser, & Peña‐Rosas, 2013; KI 1). Rather, pilots have been useful for testing the implementation of an MNP intervention before going to scale. Several pilots have shown that implementation areas considered useful to assess during a pilot were delivery model; level of coordination, training, and capacity building required; social behaviour change communication strategy; intensity of efforts; and monitoring and evaluation (GAIN, 2015; Jefferds, 2014; Jefferds, Irizarry, Timmer, & Tripp, 2013; Kodish et al., 2011; Osei et al., 2014; World Food Program, 2015; KIs 1, 9, 10, 11, 12, 14, 15, and 16).

Pilots have been helpful in generating buy‐in from local and national representatives before a larger investment is made, correcting mistakes, and uncovering issues not identified during formative research. Pilots were the most informative when designed to determine feasibility and when they included process indicators and outcome measures (e.g., coverage, appropriate use, and intake adherence) as was the case in Kenya, Mongolia, and Nepal (Jefferds et al., 2015; Mirkovic et al., 2015; Suchdev et al., 2012; Vanchinkhuu, Norov, & Bat, 2013). Examples of practice‐based learning include engaging private sector agents in expanding reach and timing the launch of MNP interventions to encourage the greatest acceptability (KIs 1, 6, 11, 12, and 16). In Kyrgyzstan, an untimely launch during the rainy season led to public's association of MNP with the increased incidence in diarrhoea (KI 6).

##### 3.1.3..4 The long‐term costs of MNP interventions as programmes scale up is usually greatly underestimated

A Copenhagen Consensus review found that micronutrient interventions were cost‐effective in general (Horton, Alderman, & Rivera, 2008). According to the review, 60 sachets of MNP every 6 months cost an estimated $1.80 per course, or $3.60 per child per year. With additional delivery costs, the total doubled to about $7.20 per child per year under a publicly funded free‐for‐product MNP model (Horton et al., 2010). A costing study of Kyrgyzstan's free‐for‐product MNP programme estimated total costs (supply, delivery, and planning) to be $8.16 per child for a 6‐month period and supply to account for 43% of total costs (Armstrong, 2009). A commercial fee‐for‐product model estimated an out‐of‐pocket cost to families of about $3.30 per course, or $6.60 per year (Bahl, Toro, Qureshi, & Shaw, 2013). Therefore, depending on the model and context, costs can vary greatly, by at least a factor of 2, depending on model, approach, and country context. Low biannual vitamin A distribution costs (less than $1 per child per year) have been used as a cost benchmark, but these interventions are not comparable in their objectives or the delivery intensity of MNP (KI 14). Nevertheless, despite the relatively high absolute costs of MNP compared to interventions such as vitamin A supplementation, Horton et al. (2008) estimate that iron‐containing MNP recover $37 for every $1 invested due to the positive effects of addressing childhood anaemia among children 6–23 months (Sharieff, Horton, & Zlotkin, 2006). It should be noted that the estimated return on investment for anaemia reduction in the Sharieff et al. (2006) analysis is based on a context with a very high anaemia rate (93%), and the total cost per child is $1.20 (or 4 months of sachets between the ages of 6 and 23 months), which includes the product and an additional 33% for delivery costs. The costs of demand creation are not estimated in any of these analyses. Key informants have reported that this return‐on‐investment data have been helpful in advocating for government funds (KIs 3 and 15).

During a tri‐country workshop involving Lao PDR, Cambodia, and Vietnam, one of the main barriers to scale‐up cited was the lack of consensus on funding. The costs—for example, additional workers and supplies—that had been seen in a pilot were challenging to meet at scale (GAIN, 2015; KIs 1 and 14). Although government funding for MNP exists in 16 of the 59 implementing countries (UNICEF, 2015), more often, interventions have been financed by external funding (KIs 1, 2, 6, 10, 11, 12, 14, 15, and 16). Full government financing of MNP has been possible in countries where funding came from replacing iron syrup interventions with MNP (Bolivia, Peru, Guatemala, and Mexico), or where large social protection programmes meant there was only a marginal cost of adding MNP (KIs 4 and 20). Increasingly, World Bank loans, social protection funds, health insurance plans, and pooled financing from government‐driven sector‐wide approach (i.e., “health basket”) budgets have been used to fund MNP interventions (KIs 1 and 3). Subsidies have defrayed some costs (KIs 2, 9, and 16). For instance Bangladesh, Madagascar, and Mozambique have adopted a free‐for‐product distribution to the most vulnerable, whereas a subsidized arm is used for others, generally those who could afford to purchase MNP (KIs 2 and 16). Experiences with free, subsidized, and full cost MNP models are presented in the delivery paper of this series (Reerink et al., 2017). Table 3 indicates some of the cost, management, and sustainability issues that have been considered in deciding between these distribution options. In many cases, long‐term costs of MNP interventions were not considered upfront, but rather funds were secured for pilots with the hope of further investments or a successful subsidized or mixed model in the future (KIs 2, 9, and 14).

**Table 3:** **Cost, management, and sustainability considerations for public versus commercial distribution options as described by key informants and literature** a

**Strategy**	**Costs**	**Management**	**Sustainability**
Fully commercial distribution (marketed through pharmacies /retail)	Advocacy, regulation, training, and other standard start‐up costs which may not be feasibly included in product price Expensive stock must be kept on hand, or consumers can become frustrated and lose interest: e.g., with ready‐to‐use therapeutic foods (Guevarra et al., 2014) Limited demand and low profit margins are a common risk and may lead to costs for social marketing/promotion The costs to the consumer, which are almost as high as the supply plus program cost of the public option	Donor support for initial start‐up costs, including supply Allow stock to be bought on consignment. Use less expensive approaches where possible. Note there is some doubt that social marketing will reduce price sensitivity (Dupas, 2014) A commercial product requires less management	Often requires donor to support countries as they start up Funds are paid back to the supplier Product price can cover the cost of marketing, or a donor is willing to fund until market is established. As long as there is demand
Subsidized	Supply and other start‐up costs Fee charged may not be sufficient to motivate coverage at desired levels, particularly for the work required and the costs of transportation and materials for demonstrations (Gittelsohn & Cristello, 2014)	Vouchers/subsidy to offset consumer costs (Siekmann, Timmer, & Irizarry, 2013); with similar products, a heavy subsidy was needed (Dupas, 2014). Study and establish level of fee required and balance against consumer price sensitivity	Wealthier consumers, government or a donor cover cost of subsidy and management Management and costs are similar to the fully commercialized model
Free (facility‐based or community‐based distribution)	Supply and other start‐up costs Staff and beneficiary time for distribution and counseling, and other expenses of distributing/ collecting (Helen Keller International, 2015) Staff time and other expenses of monitoring Budget for monthly stipend for travel costs of any outreach workers/volunteers, particularly for hard–to‐reach areas, is recommended (Helen Keller International, 2015)	Donor support for initial start‐up costs, including supply To reduce costs for both staff and beneficiary, reduce number of times distributing (whether via campaign or via regular beneficiary visits) by giving a 4‐month course twice yearly (UNICEF & CDC, 2009) and reducing number of counseling sessions (Helen Keller International, 2015) Reduce monitoring visits as capacity increases (Helen Keller International, 2015) Provide primarily for hard‐to‐reach, high‐priority target areas, and the minimum deemed feasible	Commonly requires donor to support countries as they start up Relies on government revenue (not guaranteed) to cover bi‐annual campaigns or regular health staff time and distribution Reduced number of contacts reduces opportunity to influence behavior If beneficiaries are required to collect MNP, demand may be reduced

CDC, Centers for Disease Control and Prevention; MNP, Micronutrient Powders; UNICEF, United Nations Children's Fund.

#### 3.2. Supply

##### 3.2..1 MNP has been classified as either pharmaceuticals or food related, with different implications

The regulatory classification of MNP has had implications for how the product is imported, packaged, distributed, and/or promoted (KIs 1, 2, 4, 6, 9, 10, 11, 12, 13, 16, 17, and 18). Classification as a pharmaceutical has sometimes exempted MNP from import taxation, particularly in instances where MNP has been included on the national list of essential commodities, which can also help with donor buy‐in (KIs 2, 9, 10, and 13). The pharmaceutical classification can also help increase demand due to the perceived value of medicines (Gittelsohn & Cristello, 2014). Classifying MNP as pharmaceuticals may also encourage training of pharmacists and doctors in their appropriate use (Bahl et al., 2013). However, pharmaceuticals attract stringent requirements regarding production, testing, prescribing, and dispensing, thereby limiting the number of potential suppliers and distributors, which was the case in Bangladesh (Business Innovation Facility, 2013; KIs 13, 17, and 18). Additionally, if provided through donor funding, classification may subject the procurement of MNP to particular donor rules or require special permissions (KI 21).

Classification as a food‐related product has meant greater flexibility in terms of distribution, including opportunities for a larger number of retail sales points. However, deciding on this classification has required due diligence in marketing to follow regulatory limits on health claims and promotion of the product. In particular, the International Code of Marketing of Breastmilk Substitutes and its related country‐level regulation, where applicable, has required consideration (HF‐TAG, 2013). Such local regulations are established to protect breastfeeding from competing commercial products and therefore have provisions related to labelling of infant formula and similar products for young children (WHO, 1981). The WHO does not consider MNP to be a breast milk substitute nor a food product for children (WHO, 2016). Nonetheless, many countries (Bangladesh, India, Indonesia, and Tanzania) have been cautious, seeking an exemption from the local regulations for MNP (KIs 1, 4, and 9).

##### 3.2..2 MNP has mainly been procured through international suppliers

Procurement decisions for MNP have tended to centre on quality, cost, and timing, including lead times for production and shipping (KIs 1, 2, 5, 8, 9, 10, 11, 12, 13, 14, 17, 18, 20, and 21). During planning, assessments need to estimate short‐ and long‐term volume requirements, product specifications, and the capacity of local industry to produce and store MNP (KIs 1, 5, 8, 9, 10, 11, 12, 13, 17, 18, 20, and 21). The noted advantages and disadvantages of fully imported versus locally produced sourcing are summarized in Table 4.

**Table 4:** **Considerations for imported versus local supply** a

	**Fully Imported MNP**	**Locally Packaged MNP**	**Locally Mixed & Packaged MNP**
**Description**	Procurement through a pre‐qualified international supplier, accredited by either UNICEF supply division, WFP, or a GAIN premix facility with international CoA and inspections built in	Internationally imported premix components (micronutrients / carrier / other components) and often packaging materials, locally packaged into sachets	Internationally imported premix components (micronutrients / carrier / other components) for mixing & then packaging into sachets, with either locally produced or imported packaging material
**Advantages**	Cost efficient due to scale, generally lower price per unit International companies have more resources to meet complex specification and quality requirements Consistent product quality (international standards as well as pre‐qualification of suppliers by UNICEF, WFP, GAIN) Comprehensive standardized quality control systems via internationally recognized laboratories and certificate of analysis (CoA) systems Large international suppliers may be better able to absorb fluctuations in demand (accurate forecasting challenges)	Flexible to tailor package design and marketing needs (local language, BMS code regulations in country) Local supplier may be better able to respond to program changes, providing uninterrupted, continuous supplies (Afsana et al., 2014; GAIN, 2015) Compared to local mixing option, premix supplier provides CoA, so quality control is limited to weight control and monitoring the seal/appearance May support local economic development No add‐on time from shipping and clearing	Flexible to tailor package design & marketing, as well as formulation Lower import barriers for raw material than pre‐packaged MNP or premix may reduce final price Simple equipment required for mixing May be more profitable than importing & packing premix for local companies (Guatemala) May support local economic development No add‐on time from shipping & clearing providing raw & packaging material stocks are correctly managed
**Disadvantages**	Time (6 months for production, shipping, clearing typical for re‐orders). First time procurement, registration and clearing, or non‐standard composition, packing or packaging add more time Can require large minimum orders High costs related to shipping and taxes, among other import barriers (regulations, logistics, and customs clearance at port of entry)	Required manufacturing standards and infrastructure may be hard to meet (HACCP, ISO) Local companies may not want to sell to government (often the main buyer) since they perceive government payment to be not very reliable. Has usually required significant capital investment for local company. Still likely to require importation of high‐barrier packaging materials for sachets.	In addition to the disadvantages of locally packaged MNP, also requires laboratories (internal or external) with the capacity to analyze premixes for all the relevant micronutrients and internal capacity to analyze final produce for key micronutrients on a routine basis. Introduces more complex and expensive quality control requirements and requires highly skilled laboratory personnel. Sudden significant changes in demand can only be handled by holding large stocks of raw and packing materials
**Comparative advantage**	Quality control, cost efficiency, ability to absorb large demand fluctuations	Tailored package design and marketing; shorter turnaround time for re‐orders	Potential increased profit margin for producer over packaging alone
**Example countries**	Majority of MNP‐implementing countries	Bangladesh (RENATA), Kenya, Bolivia (Laboratorios Drogueria INTI, Laboratorios Farmaceúticos LAFAR, and SIGMA Nutraceuticos)	El Salvador, Nigeria, Guatemala, China (Biomate)

a BMS, breastmilk substitutes; CoA, certificate of analysis; MNP, Micronutrient Powders; GAIN, Global Alliance for Improved Nutrition; HACCP, Hazard analysis and critical control points; ISO, https://en.wikipedia.org/wiki/International_Organization_for_Standardization; UNICEF, United Nations Children's Fund; WFP, World Food Programme.

The MNP is often sourced as fully imported finished products from centralized manufacturing operations (KIs 3, 7, 9, and 14), as this process allows for improved efficiencies and lower costs due to economies of scale (KI 5). Full importation has also led to challenges, such as high import duties, unacceptably long lead and delivery times, logistical problems at points of entry, or an outright prohibition on importation (KIs 5, 8, 9, 10, 11, 12, 13, and 14). Moreover, there have been some quality and supply interruptions with international producers. However, because international suppliers are known entities, their accountability may be higher. For example, in Cambodia, low‐quality supply resulted in the loss of a large quantity of sachets, after which international stakeholders exerted considerable pressure on the supplier to avoid a recurrence (KI 14).

Local production has been even more challenging, and only seven countries were identified as having local options for sourcing MNP (local packaging or mixing and packaging; Table 4). A major issue has been the low incentive for local suppliers to take on significant risk to upgrade manufacturing standards and/or create demand in an unpredictable market (Bahl et al., 2013). In situations where local policies are not yet adopted, demand is uncertain, and/or funding is not in place, small companies with limited capital may find the risk unappealing. Even larger companies have been reluctant to rely on government funding (KI 15). In addition, producers have needed to (a) meet prerequisites for tendering; (b) have a valid manufacturing licence; (c) be ISO 22000 compliant; (d) be hazard analysis and critical control points certified; and/or (e) be Good Manufacturing Principles certified (KIs 17 and 18). To mix and package MNP, a producer needs to rely on a certified reference laboratory to analyse incoming raw materials and have on‐site staff to perform quality control (KIs 8, 17, and 18). One major supplier indicated that the minimum volume that makes commercial sense would be an annual production of at least 100,000,000 sachets, or enough for about 833,000 children (KI 5).

##### 3.2..3 Adequate quality control systems are important for product acceptability and trust

The MNP requires both sophisticated packaging and systems to ensure stability. Before they are packaged into sachets, MNP premixes attract water from the surrounding environment; MNP also contain components that deteriorate with exposure to heat and moisture (KIs 4, 13, 17, and 18). Quality control systems ensure the quality of raw materials and the correct composition, seal integrity, packaging, and labelling of sachets (CODEX, 2005, 2013). MNP must typically be packaged in such a way as to guarantee a shelf life for 18–24 months, often under severe conditions (KI 13).

If produced according to quality standards, MNP does not affect taste or colour of foods, but a lapse in the quality control or even the continuity in the supply has had long‐lasting, detrimental effects for end users in terms of product trust, uptake, and adherence (Afsana, Haque, Sobhan, & Shahin, 2014; GAIN, 2015; Gittelsohn & Cristello, 2014). In Cambodia and Madagascar, significant discoloration and change in taste of MNP sachets were found, leading to end user refusal and, in the case of Cambodia, government suspension of delivery (KI 14). In both cases, intensified efforts were needed to restore the reputation of both MNP and the community workers promoting and delivering them (Gittelsohn & Cristello, 2014; Helen Keller International, 2015).

##### 3.2..4 Maintaining regular supply has been a challenge

Maintaining a continuous supply of MNP has been consistently reported as a challenge, due to delays in funding, current stock having to be destroyed, forecasting, supplier capacity issues, and lengthy procurement lead‐production (8–12 weeks) and shipping times (8–14 weeks) (KIs 13, 14, 15, and 20). Typically, first‐time international procurements have taken up to a year and regular resupplies can take up to 6 months, subject to procedural issues, importation, and regulatory delays, as discussed above (KI 15).

Once the product is in the community, the entity responsible for supply management has needed to ensure use before the expiry date. There have been instances in which MNP has had to be destroyed because they were not used before their expiry date or because of quality issues (KIs 9 and 14). In terms of packaging, Bangladesh and Bolivia learned through trial and error to align the numbers of sachets per package with visit frequency. For example, if community agents only visit every 2 months, providers distribute packages containing a 2‐month supply as per the recommended regimen (Helen Keller International, 2015; Lundeen et al., 2013; Schauer et al., 2017; KI 19). Finally, packaging disposal has been mentioned as an emerging concern, as the volume of nonbiodegradable sachets can be sizable. As such, Sight & Life, Scientists Without Borders and The Sackler Institute for Nutrition Science extended a challenge in 2012 to develop sustainable MNP packaging (New York Academy of Sciences, 2013).

## 4 DISCUSSION

Designing sustainable MNP interventions that reach the most vulnerable populations, especially at scale, is challenging (Zlotkin & Tondeur, 2007). In this paper, we documented experiences with the processes of planning an MNP intervention and securing supply. We captured practice‐based evidence by drawing not only on peer‐reviewed articles and programme documents but also on implementers' experiences. This review is not exhaustive, although it does cover the best documented and/or most accessible experiences. We acknowledge several limitations, including the lack of data available outside of efficacy trials, poorly documented programme experiences, and inherent issues with generalizing across contexts. The challenges in generating and distilling practice‐based learning are not specific to MNP interventions and have been noted by others (Green, 2008). Nonetheless, these findings offer lessons that can inform efforts to introduce and/or scale up MNP interventions, and those point to the need for better documentation and implementation research. We found the frameworks of Meyers et al. (2012) and Gillespie et al. (2015) useful for revealing gaps in the planning process. The application of frameworks such as these may be useful in future implementation research and planning activities.

A comprehensive MNP assessment was often not undertaken, and when they were, institutionalization, capacity building, and competencies were rarely addressed. MNP has mainly been introduced in response to the high prevalence of anaemia in young children based on the assumption that anaemia is caused by iron deficiency. The rationale for the inclusion of the other nutrients in MNP, and specifically the formulation (3, 5, or 15 micronutrients), should ideally be based on nutrient gaps identified through dietary assessments. It may be valuable to invest in local capacity for survey design, collection, and analysis of micronutrient status and dietary assessment data, and to await data from upcoming surveys, to provide current and reliable data, even if this delays start‐up. There is also a need for an established consensus on what constitutes a sufficient assessment of the appropriateness of MNP interventions in any given context, as MNP alone may not resolve anaemia if there are other associated factors, such as infections or genetics (Balarajan, Ramakrishnan, Ozaltin, Shankar, & Subramanian, 2011). If MNP is deemed appropriate on the basis of sufficient and credible data, efforts are needed to assess existing local capacity to effectively support the intervention, in the context of overall country capacity and priorities.

Promoting and supporting conducive social, political, and economic conditions for effective implementation are a continual process, which should include consideration of policy frameworks that would support MNP. The revision timelines for relevant policies, strategies, plans, or guidelines should ideally be noted early in the process; it is the role of coordinating bodies to identify and prepare for timely integration opportunities. Weaknesses in the enabling environment can cost time, money, and opportunities. Given the frequency of distribution, introducing MNP in a new setting is labour and resource intensive and requires careful planning and coordination (de Pee et al., 2007). Thus, governments may need technical and other assistance to initiate MNP interventions and avoid common pitfalls. There needs to be an overall structure for knowledge generation, leadership, and coordination of both policy and technical requirements in the initiation of an MNP intervention. Regular coordination mechanisms to oversee this process and manage and communicate MNP relevance and effectiveness are best if established early on, and pilots can be useful to test these before moving to scale.

Maintaining the core aspects of an MNP intervention and then adapting the intervention on the basis of the results of formative research and pilot studies are critical to the planning process (Zlotkin et al., 2015), and we found these types of studies were often done. In general, formative research has consistently shown high acceptability, compliance, and willingness for continued use of MNP, and pilots have indicated the potential to achieve nutritional impact (Michaux et al., 2014; Reerink et al., 2017). However, this has often not translated into successful programmatic results during the scale‐up phase (Michaux et al., 2014; Reerink et al., 2017). A better understanding of how to design formative research and pilot studies that will inform scale‐up remains a major gap. In addition, learning the types of information that can be applied from similar settings will reduce the need to replicate resource‐intensive studies.

Although IYCF programmes are the most commonly agreed‐upon platform for integrating MNP interventions, some are not functional and therefore may not warrant integration. Future guidance around MNP may consider establishing a minimum set of criteria to define a functional IYCF programme able to take on this additional task.

Long‐term financing is a major barrier to sustaining MNP interventions, especially as countries will eventually need to move from donor‐funded to government‐financed interventions. Although cost‐effectiveness has been established, funding MNP does not always take priority among other available interventions for children. The 2016 UN General Assembly resolution (70/259) proclaiming the UN Decade of Action on Nutrition from 2016 to 2025 may create more opportunities for nutrition funding and reduce country‐level financial barriers for MNP scale‐up and sustainability. A review of 100 studies spanning 32 countries concluded that a standardized method for costing micronutrient interventions would provide greater transparency to guide policymakers in developing budgets for their nutrition strategies (Fiedler, Sanghvi, & Saunders, 2008). Costing studies such as these are needed to identify how and where delivery efficiencies can be found, regardless of the delivery model (free, subsidized, or full cost). These studies can also identify geographical areas with a higher cost of delivery and/or low coverage rates to prioritize free or subsidized assistance when budgets are limited. In addition, costing studies should tease out the marginal costs of adding MNP to an ongoing programme (such as IYCF).

One of the first major decisions for MNP interventions is supply. Slowly, global policy frameworks are being built to support MNP supply. For example, to address regulatory challenges, global efforts are underway to provide specific guidance on MNP labelling in the National *Codex Alimentarius* (CODEX, 2013; Soekarjo & Zehner, 2011; Zlotkin, Siekmann, Lartey, & Yang, 2010), to mitigate the inappropriate promotion of foods for infants and young children (WHO, 2016). This may help ease MNP classification issues in the long term, and in the meantime, countries can refer to Home Fortification Technical Advisory Group tools to help ensure that local MNP marketing does not violate the Code for the Marketing of Breastmilk Substitutes (HF‐TAG, 2013).

Accessing an adequate, timely, and quality supply of MNP is crucial for a successful and sustainable MNP intervention, yet it remains one of the greatest challenges (UNICEF & CDC, 2010). The landscape for sourcing, the complexity of production, and supply chain quality control lead to a wide array of lessons learned. There is a case—where local circumstances permit—for commencing with a fully imported product and moving to local production at a later stage. Long‐term cost savings of local production have not been fully evaluated. The development and implementation of quality control systems for both supply and distribution of MNP need to be considered at start‐up, as errors can negatively impact acceptance and proper use. Aside from the obvious benefits of quality control, supply of MNP benefit from integration into existing delivery systems to reduce costs and improve efficiencies in the long term.

Although anecdotal information exists about MNP processes around planning, coordination, and reliable supply, further research is needed to generate wider knowledge and overall efficiency. Priority research areas or tools/resources identified during the consultative process included the following:

- Identify the “tipping points” to achieving government buy‐in (political and financial) of MNP and related contextual factors.
- Establish benchmarks for the level of coordination and capacity building needed to manage the different efforts that MNP interventions may require.
- Develop basic formative research questions, methodologies, and tools that programmes can use to inform the programming of and communication around MNP, focusing on the context‐specific knowledge, attitudes, and behaviours around complementary feeding and other aspects that might impact adoption.
- Identify best practices to build MNP funding into scale‐up plans and national programmes.
- Articulate decision‐making pathways to source MNP according to capacity requirements and regulatory and import tariff regimes.
- Analyse the production process and timelines (e.g., packaging formats and shelf life, ordering, and delivery time windows) from a systems perspective to improve continuous supplies.
- Assess cost savings or economic benefits of local production.
- Analyse cost‐effectiveness of various integrated options within health and nutrition policies to provide quick and reliable estimates for rollout of MNP interventions and their sustainability.

## CONFLICT OF INTEREST

CS, CHM, CND, SW, GT, RDWK and SZ declare no conflicts of interest. NS consults for Global Alliance for Improved Nutrition (GAIN) and for a number of South African and global companies involved in staple food fortification and the supply of micronutrients.

## CONTRIBUTIONS

CS, CHM, NS, and CND did the primary drafting of this paper. RDWK, GT, SW, RT, and SZ contributed to the analysis and interpretation of the data as well as revising the draft critically. RDWK chaired the working group, and CHM served as secretariat. All authors contributed to the conception and design of the paper, and reviewed and approved the overall paper.

## Supporting information

Data S1. Supporting info item
