# Supplementary material for: Experiences and lessons learned for planning and supply of micronutrient powders interventions
Source: Matern Child Nutr. 2017 Sep 29;13(Suppl 1):e12494. doi: 10.1111/mcn.12494 (PMC5656916; doi:10.1111/mcn.12494)
Supplement: Supplementary file 1 — Data S1. Supporting info item [file MCN-13-e12494-s001.doc]

**Supplementary Material 1**

**Working Group 1 Questionnaire on Planning and Supplies for Key Informant Interviews (formatted as presented to KIs)**

| **Questionnaire: Planning and Supplies Working Group** |
| --- |
| - *Your responses to this questionnaire will be used to share your experiences and insights on micronutrient powder programming across the three working groups for the upcoming MNP Consultation. Please fill out the form by October 12.* - *Do not feel required to answer every question, but please do at least skim the entire questionnaire and focus on "specific identifiable lessons learned" -- positive or negative -- that you would like to share. If you have already been interviewed by a group, you do not need to respond to that group's questionnaire.* - *Before completing this questionnaire, feel free to solicit input from colleagues past and present and others in your country or office who you think would have useful responses. Multiple perspectives from the country or office can be rolled into one response form.*   *Name: _________________________*   - *This will only be used to ensure that we can follow up with you about any questions. We will not use your name to attribute any answers without first receiving your permission.*   *In what country(ies) do you have experience working with MNP programs?*  *Please note if you have any requests for anonymity of a particular country experience* |
| **Creating an Enabling Environment** |
| ***Stakeholder Sensitization*** |
| 1. How did the country arrive at a decision to introduce MNP? |
| 1. What evidence was most useful in the decision-making process? |
| 1. Who was targeted for advocacy? On what topics? What stakeholders were instrumental in advocating or being advocated to? |
| 1. Was the cost-effectiveness considered and/or presented, (of MNP relative to other interventions a country could use to address anemia)? If so, to whom was it presented and how? |
| 1. Was the effectiveness/efficacy evidence considered and/or presented (of MNP relative to other interventions a country could use to address anemia)? If so, to who was it presented and how? |
| 1. How did you assess the needs, interests, resources, and contributions of each stakeholder (including government entities)? |
| 1. What were the greatest challenges and/or barriers faced in creating an enabling environment for MNP? Within the health sector? Within the nutrition sector? Within other sectors? How were these overcome or do they continue to be challenges? |
| 1. Was MNP formulation an issue in creating an enabling environment? If so, how? |
| 1. How if at all did advocacy differ from start up, to scale up, to trying to establish sustainability? What kind of continued advocacy and/or reporting is needed to maintain an enabling environment for MNP within the country? |
| ***Integration of MNP into relevant policies and programs*** |
| 1. What needs to be in place or considered to allow for MNP programs (IYCF programs, national nutrition strategies, etc.)? |
| 1. What if any do you believe is the 'cost savings' of integrating into existing programs? E.g., leveraging monitoring systems, etc. |
| 1. Are MNP part of infant and young child feeding policies and programs or are they stand-alone? What specific policy or program guidance is provided on whether and how to integrate MNP into existing programs (e.g., IYCF, anemia reduction, supplementation such as vitamin A, or industrial fortification)? |
| 1. When coordinating or integrating with complementary efforts, what is the mix of interventions and why? |
| 1. What are the entry points to get MNP into national nutrition policies? |
| 1. How have you gotten MNP included in monitoring systems (e.g., community-based information systems, electronic health information systems, others)? |
| 1. What guidance, if any, is given on the role of MNP within the mix of nutrition interventions and products addressing micronutrient and other deficiencies (e.g. LNS, RUSFs, micronutrients in pregnancy, etc)? |
| ***Regulatory Processes*** |
| 1. How were MNPs classified (e.g., as a drug, food additive, fortificant, etc.) to smooth registration, importation, manufacturing, and/or labeling? |
| 1. What, if any, issues regulated the registration, importation, manufacturing, packaging, and/or labeling of MNPs? |
| 1. How do MNPs fit within regulations governing the international Code of Marketing of Breastmilk Substitutes? |
| **National Level Coordination of MNP Planning and Programs** |
| ***Strategic Planning & Financing*** |
| 1. What was the short-term vision for how MNP would be implemented in the country? The long-term vision? |
| 1. How are MNPs financed in the country, and to what extent, if any, is budgetary support included in the national or district budgets? (e.g., are MNP integrated into national health systems, so personnel costs covered? Or is the program externally funded and implemented)? If not covered by government, what would it take to make the government absorb costs? |
| 1. Is there long-term sustainable funding for MNP? If so, what and how was this accomplished? |
| 1. Do decentralized health systems have an impact on implementation? What levels of support are needed and for what purposes at national and district levels? What other resources are needed for MNP in the country? |
| 1. How are resources being mobilized for scale-up? How could they be mobilized? |
| ***Landscape Analyses*** |
| 1. What was the initial (or current) rationale for establishing target areas, target groups, and approach? |
| 1. Was a landscape analysis undertaken to identify stakeholders' needs, interests, and possible contributions to an MNP program?   *If no, move on to question number 32. If yes, please answer questions 27-31.*  Yes 🞎  No 🞎 |
| 1. What were the findings related to addressing issues on sustainability, institutionalization, capacity building, and competencies within the health system? |
| 1. What were the findings related to identifying partners to approach for the implementation of specific program activities, such as behavior change and monitoring? |
| 1. What were the findings related to identifying and resolving potential disagreements or conflicts of interest which might arise, such as catchment areas or other programming already in place? |
| 1. What were the findings related to identifying and capitalizing on opportunities and relationships throughout design and implementation? |
| 1. What were the findings related to identifying the best platform or combination of platforms to deliver MNP? |
| ***Coordinating bodies*** |
| 1. Who are the stakeholders and key groups at the national, district, and community levels and what roles do they play with regards to the MNP program? |
| 1. Do the groups coordinate? If so, how and on what specific issues and at what levels (i.e. national, district)? |
| 1. Who if anyone provides regular updates? Course correction? |
| 1. Is there a centralized coordinating body specific for MNP as part of an existing oversight or coordinating body? Or is there a separate/parallel coordinating body? |
| 1. What kind of feedback is provided to the oversight committee? At what frequency? If little or no feedback is provided, why and what kind of information do you feel is important for that body to obtain? |
| **Ensuring Regular, Reliable, and Constant Supply at National and District Level** |
| ***Overall supply issues*** |
| 1. How did you address any supply chain issues, either with private sector or public sector? What kind of problems did you encounter (planning, procurement, importation, registration, distribution, promotion, forecasting etc) and how were they resolved? |
| ***Decision making about procurement vs. production/packaging of MNP*** |
| 1. How often have you worked on MNP products that were a) Importation of fully packed sachets vs. b) Local packing of imported premix vs. c) Local mixing AND packing of imported premix? |
| 1. What are the pros and cons of each? |
| 1. What regulatory issues (import controls, tariffs, compulsory local content etc.) have you seen impact these decisions (fully packed, vs. local packing, vs. local mixing AND packing)? |
| 1. What else has influenced your choice of a vs. b vs. c? |
| 1. What should be the main focus areas for any MNP sourcing / manufacturing feasibility study? |
| ***Procurement of packed MNP:***  *IF YOU HAVE WORKED ON "a." (FULLY PACKED IMPORTED), Please answer Questions 43-50. If you have not, move to the next section, question number 51.* |
| 1. What factors influenced choice of source country / supplier? |
| 1. Was an off-the-shelf option available from the supplier or was it customized? If so, how? |
| 1. How did you establish the acceptability of the suppliers' quality & safety systems? |
| 1. What lessons did you learn in the process of purchasing and importing the product? |
| 1. Was there a quality testing system contemplated prior the product's arrival? Can you describe what you subsequently did in this regard (especially after product arrival in country and as heading out to distribution points)? |
| 1. How did you manage inspection of the incoming product? E.g., what procedures did you apply if the incoming product did not meet specification? |
| 1. How did you handle and store the imported product? |
| 1. How did you manage and control for the "use by" or "best buy date"? |
| ***Production of MNP (packing imported premix and/or mixing and packing)***  *IF YOU HAVE WORKED ON "B Or C." (PRODUCT THAT INVOLVED PACKING OR MIXING AND PACKING, IMPORTED PREMIX): Please Answer Questions 51-60. If you have not, move to the next section, question number 61.* |
| 1. Did you modify existing premises or did you go the 'greenfield' route (build a plant from the beginning)? |
| 1. What challenges, if any, did you face in ensuring the facility met safety standards? |
| 1. How did you calculate your estimates of volume requirement? How, if at all, did you make provision for future increases in volume? |
| 1. How did you select packing machinery? Did you use single or multi-lane machinery? |
| 1. What constraints did you face in sourcing skilled maintenance labor for the machines? |
| 1. What routine quality control checks and food safety monitoring systems were put in place? |
| 1. What are the quality assurance and/or quality control issues faced in the production, mixing, and/or packing of MNP? |
| 1. How did you handle any requirements for local analysis of micronutrient content in premixes, if any? |
| 1. What kind of external inspection and monitoring did you face from local authorities, if any? |
| 1. Did any of your projects involve mixing as well as packing? If yes, why? |
| ***MNP supply management systems*** |
| 1. What lessons did you learn about storage, transport, and delivery to distribution sites? |
| 1. What, if any, problems did you have distributing MNP down to the community and HH level? |
| 1. What delivery channels did you use (public, private, mixed)? [We do not need to know about choosing a channel or the experience with a channel] |
| 1. For a given channel, how did your supply management systems function? What lessons did you learn? |
| 1. Within the supply management system, were there inspections at different stages? Were there any violations of the system and if so, what if any enforcement mechanism was used? |
| 1. How did you handle waste management of sachets, if that was raised in your project? |
